# Expression of Concern on “Benefits of *Zataria multiflora Boiss* in Experimental Model of Mouse Inflammatory Bowel Disease”

**DOI:** 10.1155/ecam/9868640

**Published:** 2026-08-20

**Authors:** Evidence-Based Complementary and Alternative Medicine

EXPRESSION OF CONCERN: L.A. Nakhai, A. Mohammadirad, N. Yasa, et al., “Benefits of *Zataria multiflora Boiss* in Experimental Model of Mouse Inflammatory Bowel Disease”, *Evidence-Based Complementary and Alternative Medicine* 4, no.1 (2006): 43–50, https://doi.org/10.1093/ecam/nel051.

This Expression of Concern is for the above article, published online on 19 June 2006 in Wiley Online Library (https://wileyonlinelibrary.com), and has been issued by John Wiley & Sons Ltd.

The Expression of Concern has been issued due to the investigation of concerns flagged by a third party on PubPeer [1]. The concerns relate to the similarity of the histopathology images of the animals from the control and prednisone groups to images in multiple publications by the same author group [2, 3]. Although the description of the images is identical between the papers, this was not disclosed. Additional concerns were identified related to the similarity of the microscopic scores reported in the three papers. However, the journal could not obtain the raw data from the authors due to the article’s age and could not definitively establish that the above‐mentioned concerns affect the overall conclusions of the article. Therefore, the journal has decided to issue an Expression of Concern to inform and alert readers. The authors have been notified.
